# Comparison of Four Protocols to Generate Chondrocyte-Like Cells from Human Induced Pluripotent Stem Cells (hiPSCs)

**DOI:** 10.1007/s12015-016-9708-y

**Published:** 2016-12-16

**Authors:** Wiktoria Maria Suchorska, Ewelina Augustyniak, Magdalena Richter, Tomasz Trzeciak

**Affiliations:** 1 Radiobiology Lab, Greater Poland Cancer Centre, 61- 866 Poznan, Poland; 2 Department of Electroradiology, Poznan University of Medical Sciences, 61-866 Poznan, Poland; 3 The Postgraduate School of Molecular Medicine, Medical University of Warsaw, 02-091 Warsaw, Poland; 4 Department of Orthopedics and Traumatology, Poznan University of Medical Sciences, 61-545 Poznan, Poland

**Keywords:** Human induced pluripotent stem cells, Cell differentiation, Chondrocytes, Regenerative medicine

## Abstract

Stem cells (SCs) are a promising approach to regenerative medicine, with the potential to treat numerous orthopedic disorders, including osteo-degenerative diseases. The development of human-induced pluripotent stem cells (hiPSCs) has increased the potential of SCs for new treatments. However, current methods of differentiating hiPSCs into chondrocyte-like cells are suboptimal and better methods are needed. The aim of the present study was to assess four different chondrogenic differentiation protocols to identify the most efficient method of generating hiPSC-derived chondrocytes. For this study, hiPSCs were obtained from primary hu﻿man dermal fibroblasts (PHDFs) and differentiated into chondrocyte-like cells using four different protocols: 1) monolayer culture with defined growth factors (GF); 2) embryoid bodies (EBs) in a chondrogenic medium with TGF-β3 cells; 3) EBs in chondrogenic medium conditioned with human chondrocytes (HC-402-05a cell line) and 4) EBs in chondrogenic medium conditioned with human chondrocytes and supplemented with TGF-β3. The cells obtained through these four protocols were evaluated and compared at the mRNA and protein levels. Although chondrogenic differentiation of hiPSCs was successfully achieved with all of these protocols, the two fastest and most cost-effective methods were the monolayer culture with GFs and the medium conditioned with human chondrocytes. Both of these methods are superior to other available techniques. The main advantage of the conditioned medium is that the technique is relatively simple and inexpensive while the directed method (i.e., monolayer culture with GFs) is faster than any protocol described to date because it is does not require additional steps such as EB formation.

**Electronic supplementary material:**

The online version of this article (doi:10.1007/s12015-016-9708-y) contains supplementary material, which is available to authorized users.

## Introduction

Human articular cartilage (AC) has a poor capacity for self-regeneration, which is why joint disease—typically characterized by cartilage degradation—is a serious condition often only amenable to surgical interventions. Stem cells (SC) are a highly promising cell replacement therapy to treat cartilage degradation in common articular diseases such as osteoarthritis [1–3]. Cell-based cartilage tissue engineering for cartilage regeneration [4] is considered a true breakthrough in the field of SC research, as shown in the research carried out by Takahashi and Yamanaka, who obtained pluripotent cells from human fibroblasts [5, 6]. Defined transcription factors can be used to transform terminally-differentiated cells into human induced pluripotent stem cells (hiPSCs), giving rise to SCs that share a wide range of characteristics with human embryonic SCs (hESCs) [7].

Numerous techniques for chondrogenic differentiation of hiPSCs are available, including micromass culture, directed differentiation, pellet culture, and embryoid body (EB) formation (the most common method) [8]. However, all of these techniques have shortcomings, particularly with regards to their efficiency in differentiating hiPSC cells into chondrocyte-like cells [9, 10]. Thus, it is important to emphasize that, in an attempt to develop a faster and more cost-effective method, our group developed a novel directed differentiation technique involving the use of a monolayer culture with added growth factors (GFs). The advantage of this approach is that no additional steps (e.g., EB formation) are required and, moreover, only relatively small amounts of GFs are needed.

The main aim of this study was to evaluate and compare four different hiPSC chondrogenic differentiation protocols to identify the most efficient and least expensive method(s) of generating chondrocyte-like cells. We evaluated the following methods: 1) monolayer culture with the addition of defined GFs (DIRECT protocol), 2) EBs differentiated in chondrogenic medium with TGF-β3 cells (TGF-β3 protocol) [modified 11], 3) EBs differentiated in chondrogenic medium conditioned with human chondrocytes (HC-402-05a cell line) (COND protocol), and 4) EBs differentiated in chondrogenic medium conditioned with human chondrocytes and supplemented with TGF-β3 (TGF-β3+ COND protocol).

Our findings indicate that hiPSCs can be successfully differentiated into chondrogenic lineages using all four of these protocols. The cells obtained with all of these methods presented the characteristics of mature chondrocytes. We found that both the conditioned medium (COND protocol) and the DIRECT protocol allowed us to obtain chondrocyte-like cells quickly and inexpensively. Furthermore, the novel directed differentiation protocol produced chondrocyte-like cells whose properties were comparable to cells differentiated through the better-known approach of EB formation in the presence of TGF-β3. The use of this new method could help to reduce the time and expense of producing cells for use in regenerative medicine. This study contributes to a greater understanding of the role of hiPSCs in regenerative medicine and should help to further develop fully functional chondrocytes derived from SCs.

## Materials and Methods

### Culturing Human Induced Pluripotent Stem Cells

We seeded the hiPSCs—obtained through a reprogramming process described previously [12] —on 10-cm Petri dishes in Matrigel (Becton Dickinson) coated previously with inactivated murine embryonic fibroblasts (MEFs) as a feeder layer (1 × 10^6^). After 24 h, the hiPSCs were seeded at 2 × 10^6^ in hiPSC growth medium consisting of the following: DMEM F12 with L-Glutamine (Merck Millipore, Germany); 20% KSR (Thermo Fisher Scientific, MA, USA); 1% NEAA (Merck Millipore, Germany); 0.1 mM β-mercaptoethanol (Merck Millipore, Germany); and 1% P/S (Merck Millipore, Germany). Prior to use, the medium was supplemented with fibroblast growth factor 2 (FGF-2) (10 ng/ml) (Merck Millipore, Germany). The culture medium was changed daily.

### Chondrogenesis in Vitro

We used a standard chondrogenic medium, as follows: DMEM F12 with L-Glutamine; 10% FBS (Biowest, France); 50 μM L-proline (Sigma Aldrich, MO, USA); 50 μM ascorbic acid (Sigma Aldrich, MO, USA); 1 mM sodium pyruvate (Biowest, France); 1% ITS + Premix (Corning, NY, USA); 1% P/S and 10^−7^ M dexamethasone (Sigma Aldrich, MO USA).

### Chondrogenesis via Monolayer Culture (DIRECT Protocol)

**Fig. 1:**
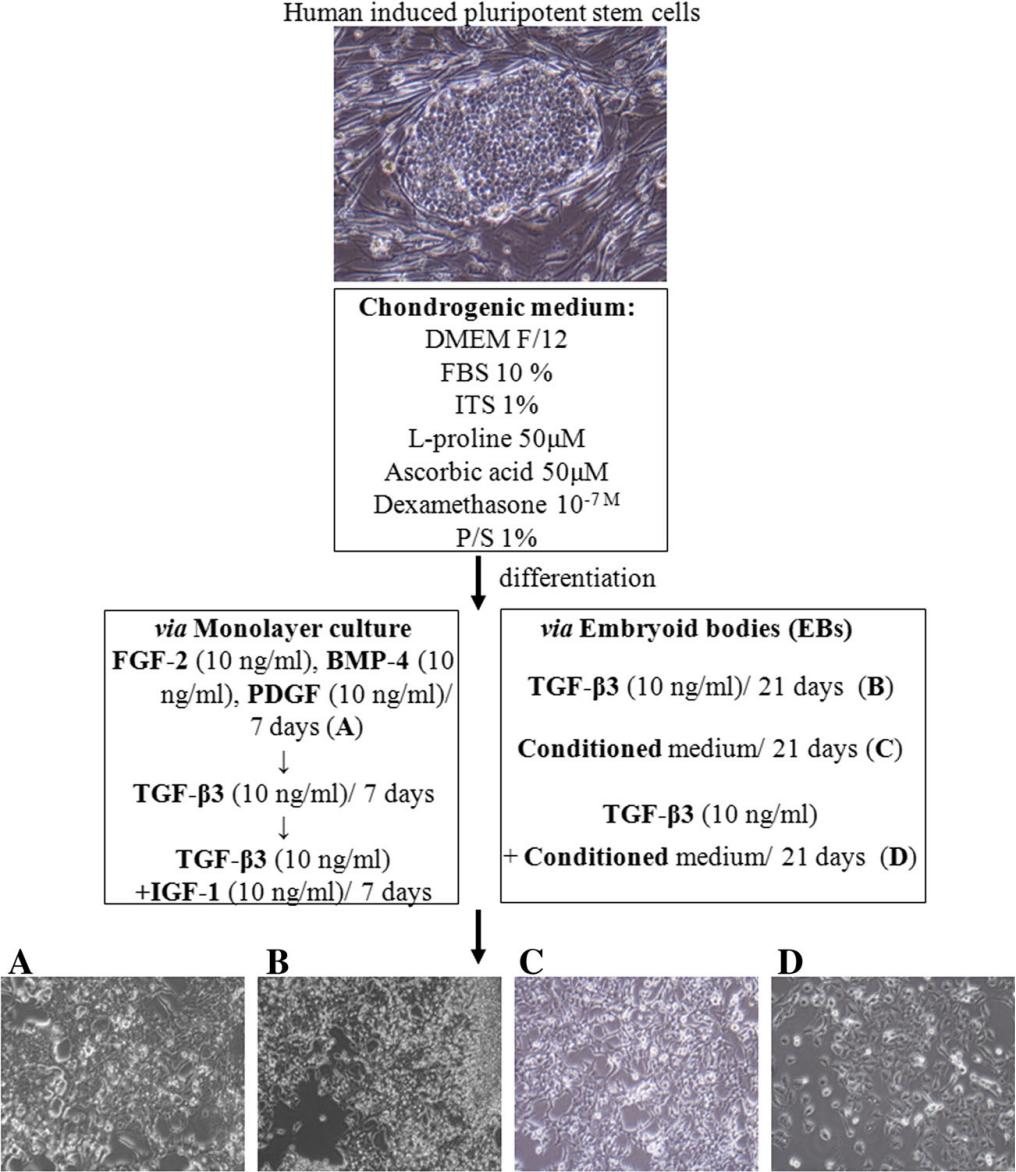
HiPSCs were differentiated through four different protocols: **a** monolayer culture with a set of defined growth factors (GF) (DIRECT protocol); **b** embryoid bodies (EB) with the exogenous addition of TGF-β3 (TGF-β3 protocol); **c** EBs in a medium conditioned on the HC-402-05 cell line (COND protocol); and **d** EBs in a conditioned medium supplemented with TGF-β3 (TGF-β3+ COND protocol). In all cases (**a**, **b**, **c**, **d**), the cells obtained through these differentiation processes presented morphological characteristics of chondrocytes

Firstly, hiPSCs were cultured for 1 week in a pro-mesodermal medium consisting of the following: DMEM F12 with L-Glutamine, 10% FBS supplemented with FGF-2 (10 ng/ml); BMP-4 (10 ng/ml) (ImmunoTools, Germany); and PDGF-BB (10 ng/ml) (PeproTech, UK). Then, the medium was replaced with a standard chondrogenic medium supplemented with TGF-β3 (10 ng/ml) (ImmunoTools, Germany) for another week. After that, the medium was replaced with a chondrogenic medium supplemented with TGF-β3 (10 ng/ml) and IGF-1 (10 ng/ml) (Peprotech, UK) for one final week of enhanced chondrogenesis in vitro (DIRECT protocol; Fig. 1a)

### Embryoid Body Formation

At 80% confluency, hiPSC colonies were passaged and dissociated into clumps with 0.1% collagenase IV solution (Thermo Fisher Scientific; Massachusetts, USA). The cells were centrifuged to remove collagenase and transferred into non-adherent 96-well plates (1000 cells per well) (Brand, Germany) in EB growth medium (a hiPSC growth medium without FGF-2). The EBs formed within 24 h and were visible as free-floating aggregates. The culture medium was changed every 48 h. On day 7 the EBs were used for chondrogenic differentiation.

### Medium Conditioning

A standard chondrogenic medium was used for conditioning: DMEM F12 with L-Glutamine; 10% FBS; 50 μM L-proline; 50 μM ascorbic acid; 1 mM sodium pyruvate; 1% ITS + Premix; 1% P/S; and 10^−7^ M dexamethasone. This conditioning medium was used for the HC-402-05a cell line. After 24 h, the conditioned medium was collected and administered to the differentiated EBs.

### Chondrogenesis Using Embryoid Bodies

The mature EBs were transferred onto 6-well plates (10 EBs per well) previously coated with 0.1% gelatin (Merck Millipore, Germany) and allowed to adhere for the next 24 h. After 24 h, the medium was replaced with one of the following: 1) a chondrogenic medium supplemented with TGF-β3 (10 ng/ml) (TGF-β3 protocol; Fig. 1b), considered to be the GF with the most chondrogenic potential; 2) a chondrogenic medium conditioned on the HC-402-05a cell line (COND protocol; Fig. 1c or 3) a chondrogenic medium conditioned on the HC-402-05a cell line supplemented with TGF-β3 (10 ng/ml) (TGF-β3+ COND protocol; Fig. 1d). The positive effect of standard chondrogenic medium containing exogenous TGF-β3 (10 ng/ml) on pluripotent SCs was previously tested and confirmed in our laboratory [13]. The chondrogenic medium was changed every 48 h. The total culturing period was 21 days. In all analyses, a stable cell line of adult human articular chondrocytes (HC-402-05a) served as a positive control based on the recommendations of the European Collection of Authenticated Cell Cultures (ECACC) for evaluation of the differentiation process in in vitro model systems (https://www.pheculturecollections.org.uk/products/celllines/primarycells/detail.jsp?refId=06090702&collection=ecacc_nepc).

### Reverse Transcriptase-PCR and Real-Time PCR

Total RNA was extracted from cells with Trizol (Sigma Aldrich, MO, USA). One μg of total RNA per 20 μl reaction volume was reverse-transcribed using the iScript™ cDNA Synthesis Kit (Bio-Rad, CA, USA). Real Time-PCR reactions were performed using the LightCycler® 480 Probes Master (Roche, Switzerland) and the appropriate probe for each primer. cDNA samples were analyzed for genes of interest and for the reference gene GAPDH (05-190-541-001, Roche Diagnostics, Switzerland), selected based on recently published data on chondrogenic differentiation of hiPSCs [14]. The expression level for each target gene was calculated as -2^ΔΔct^. The reaction was performed in triplicate for the gene of interest. The real-time polymerase chain reaction for individual genes expression analysis was carried out using LightCycler 96 with specific primers (S Tab. 1) designed with Universal Probe Library software (Roche Diagnostics, Switzerland).

### Immunofluorescence Analysis

The cells were transferred into a 0.1% gelatin-coated 48-well plate for 48 h and then washed with phosphate buffered saline (PBS) (Sigma Aldrich, MO, USA) and fixed for 20 min in 100% methanol (intercellular antigens) (CHEMPUR, Poland) or 4% formaldehyde (extracellular antigens) (CHEMPUR, POLAND) (400 μl of methanol/formaldehyde per well). Then, the cells were rinsed with PBS containing 1% bovine serum albumin (BSA) (Sigma Aldrich, MO, USA) and incubated for 30 min in PBS containing 1% BSA and 0.2% Triton X-100 (Sigma Aldrich, MO, USA). After 30 min, the cells were washed with PBS containing 1% BSA. The primary antibodies were diluted in PBS containing 1% BSA and 0.2% Triton X-100 and the cells were incubated overnight at 4 °C with the following primary antibodies (all from Abcam PLC, U.K.): COMP (1:100) (ab128893) ; type II collagen (1:100) (ab34712); type IX collagen (1:100) (ab134568); aggrecan (1:85) (ab3778); SOX6 (1:50) (ab30455); SOX9 (1:50) (ab59252); E- cadherin (ab152102) as well as Nanog (1:50) (MABD24) and OCT3/4 (1:50) (MABD76) (both from Merck Millipore, Germany). After conjugation with the primary antibodies, the cells were rinsed three times with PBS containing 1% BSA. The following secondary antibodies were diluted with 1% BSA in PBS and incubated in the dark for 1 h at 37 °C: mouse monoclonal anti-IgG (715-545-150), mouse monoclonal anti-IgM (715-545-140), and rabbit polyclonal antibody (1:500) (711-546-152) (Jackson ImmunoResearch, PA, USA). After the cells were washed three times with 1% BSA in PBS, they were stained for 5 min with diamidino-2-phenylindole dye (DAPI) (Sigma Aldrich, MO, USA) solution in water (1:10000) and then washed with PBS before undergoing microscope analysis. Signal intensity was evaluated using the ImageJ bioinformatics programme (v. 1.49j; Wayne Rasband, NIH, USA).

### Flow Cytometry Analysis

Cells were stained for TRA-1-60, TRA-1-81, CD44, and CD151 with the anti- human TRA-1-60 (14-8863-82), anti-human TRA-1-81 (14-8883-82), anti-human/mouse CD44 (14-0441-86), and anti-human CD151 (16-1519-85) antibodies, respectively, according to the manufacturer’s instructions (all from eBioscience, CA, USA). Briefly, approximately 5 × 10^5^ differentiated and control cells were collected. The cells were rinsed with PBS and incubated for 30 min at 4 °C in PBS containing 1% BSA with primary antibodies (0.5 μl per 100 μl sample). Then, the cells were rinsed with PBS containing 1% BSA and incubated with the secondary biotinylated antibodies (anti-mouse IgM Biotin, 13-5790-85; anti-mouse IgG Biotin, 13-4013-85; and anti-rat IgG Biotin, 13-5790-85; all from eBioscience, CA, USA) (0.5 μl per 100 μl sample) in PBS containing 1% BSA for 30 min at 4 °C. Finally, the cells were rinsed and stained with streptavidin with APC conjugated (17-4317-82) (0.3 μl per 100 μl sample) (eBioscience, CA, USA) for 15 min at room temperature. The stained cells were rinsed twice, resuspended in 500 μl in 1x CellFIX (Beckton Dickinson, NJ, USA) and analyzed with a flow cytometer (BD Accuri® C6) using a 675/25 FL4 filter within 1 h. Fluorescence intensity in arbitrary units was plotted in histograms. Data were analyzed using FlowJo software (FlowJo, LLC; OR, USA).

### Statistical Analysis

All experiments were performed at least three times. Results are reported as means ± standard deviation. Comparisons between the study groups and controls were performed using the unpaired Student’s t-test. Statistical tests were performed with GraphPad Prism (version 5.0a, GraphPad Software, Inc., San Diego, CA). *P* values <0.05 were considered significant.

## Results

All cells differentiated into chondrogenic lineage via the four differentiation techniques lost morphology assigned to pluripotent SCs and acquired features characteristic for chondrocytes. In all cases, the differentiated cells were unable to form colonies, and the cells were long and spindly and characterized by enhanced production of extracellular matrix (Fig. 1a, b, c, d). After differentiation, the chondrocyte-like cells were subjected to further analyses.

### Expression of Pluripotency and Chondrogenic Genes in the Chondrocyte-Like Cells

**Fig. 2:**
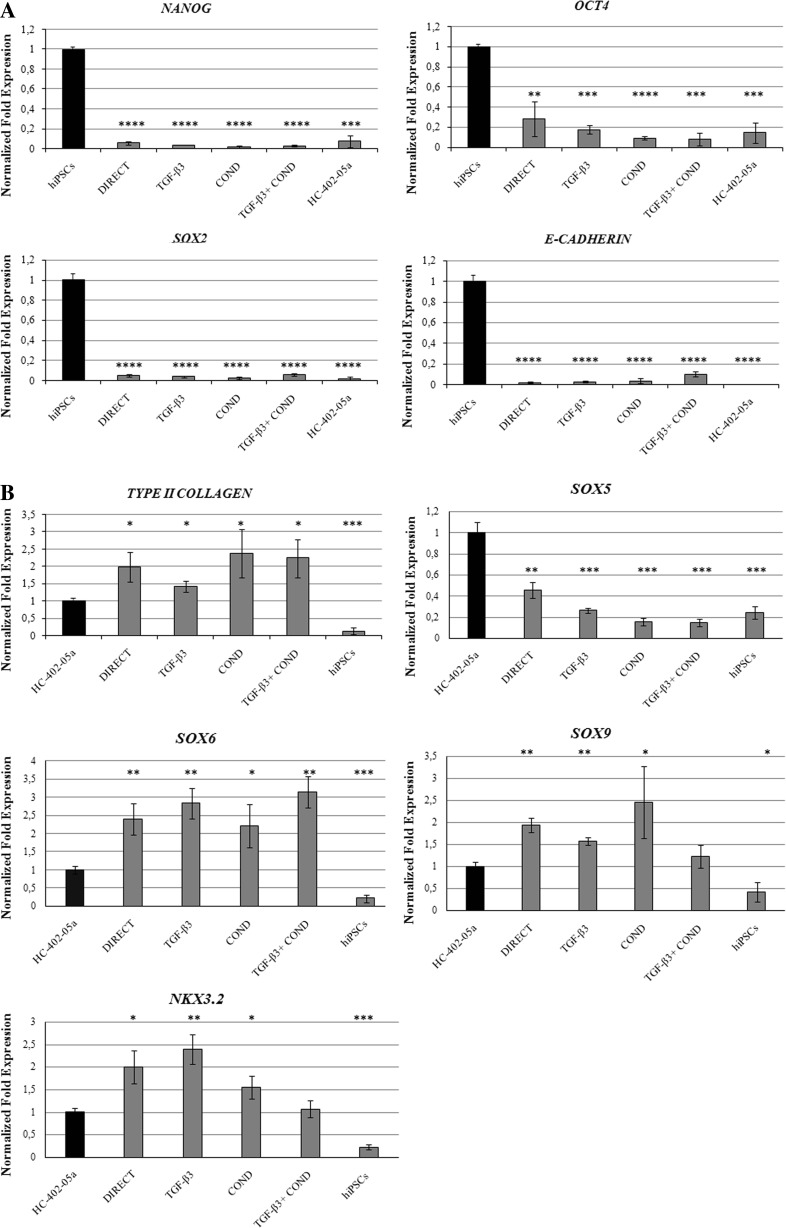
The human-induced pluripotent stem cells (hiPSC)-derived chondrocytes: DIRECT, TGF-β3, COND and TGF-β3 + COND were analyzed by qPCR. The cells did not express *Nanog*, *OCT4*, *SOX2,* or *E-cadherin* **a**, the genes responsible for maintaining pluripotency. The differentiated cells expressed chondrogenic genes: *type II collagen*, *SOX5*, *SOX6*, *SOX9* and *NKX3.2* **b**

The pluripotency genes *Nanog*, *octamer-binding transcription factor 4 (OCT4)*, *(sex determining region Y)-box 2 (SOX2)*, and *E-cadherin* were not expressed in any of the differentiated cells obtained through the four differentiation protocols, although these genes were expressed in the original hiPSCs (Fig. 2a). All hiPSC-derived chondrocytes expressed the chondrogenic genes *type II collagen*, *(sex determining region Y)-box 5,6,9 (SOX5*,*6*,*9),* and *NK3 Homeobox 2 (NKX3.2)* at variable levels (Fig. 2b). Expression of *type II collagen* was approximately twice as high in the differentiated cells versus controls (HC-402-05a cell line). Expression of *SOX Trio* genes (*SOX5, SOX6* and *SOX9*) was variable, with *SOX5* expressed in all investigated cells, but at lower levels than in human articular chondrocytes. However, both *SOX6* and *SOX9* were expressed at higher levels in the chondrocyte-like cells compared to the positive controls. The mRNA assigned to *NKX3.2* was detected in all differentiated cells, but most prominently in those obtained via the DIRECT and TGF-β differentiation methods.

### Immunofluorescence Analysis of hiPSC-Derived Chondrocytes

**Fig. 3:**
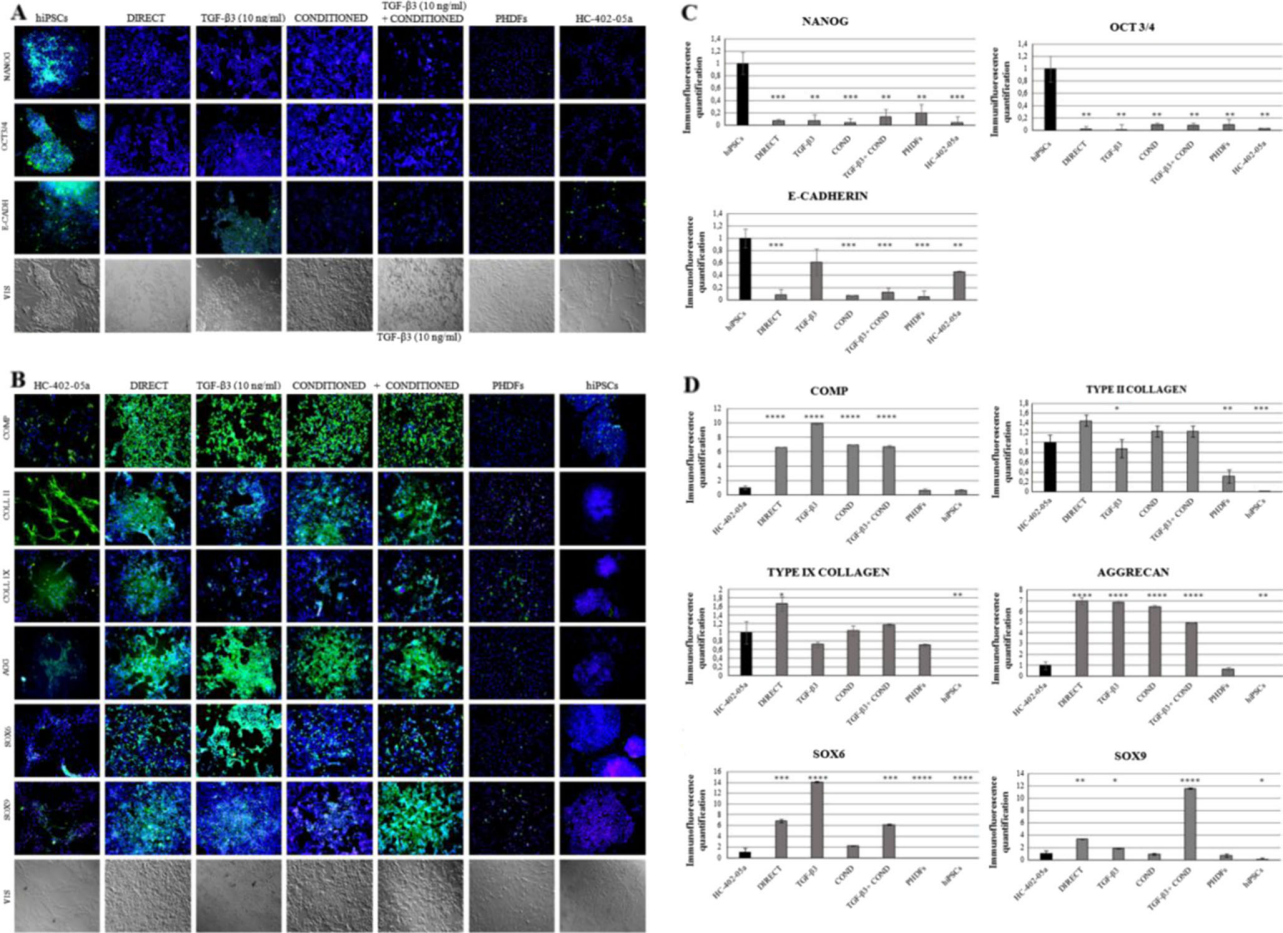
The presence of chondrogenic markers was demonstrated by immunofluorescence analysis. The human-induced pluripotent stem cells (hiPSC)-derived chondrocytes: DIRECT, TGF-β3, COND, TGF-β3+ COND did not reveal the presence of pluripotency markers: Nanog, OCT4, E-cadherin (**a**), but they did express desirable chondrogenic proteins: COMP, type II and IX collagen, aggreccan (AGG), SOX6 and SOX9 (**b**). The pluripotency and chondrogenic markers were quantified to better show the differences between particular differentiation protocols (**c** and **d**)

The results obtained at the mRNA level were confirmed at the protein level by immunofluorescence analysis. Immunofluorescence staining proved the lack of pluripotency markers *Nanog, OCT3/4,* and *E-cadherin* in most differentiated cells. The only cells, which expressed *E-cadherin* were those differentiated in the chondrogenic medium with TGF-β3 (TGF-β3 protocol) (Fig. 3a, c). The differentiated cells showed the presence of the following chondrogenic markers: cartilage oligomeric matrix protein (COMP), type II collagen, type IX collagen, aggrecan, SOX6 and SOX9. COMP production was high in all the cells obtained through in vitro chondrogenesis. Type II collagen was present in all differentiated cells at levels that were similar to those observed in the control cell line (HC-402-05a cells). Type IX collagen was detectable in all differentiated cells, with the highest levels observed in DIRECT cells (at higher levels than seen in human articular chondrocytes). All the cells obtained by all four protocols showed very high production of aggrecan, which was at higher levels than observed in the HC-402-05a cell line. SOX6 was present in all the cells after chondrogenic differentiation, with extremely high levels in TGF-β3 cells and high levels in the DIRECT and TGF-β3+ COND cells. SOX9 was produced by all differentiated cells, with the highest production in TGF-β3+ COND cells. We did not observe any dedifferentiation of hiPSCs: markers present in all differentiated cells differed significantly from those expressed by primary human dermal fibroblasts (PHDFs) (Fig. 3b, c).

### Flow Cytometry Analysis of Pluripotency and Chondrogenic Proteins in Differentiated Cells

**Fig. 4:**
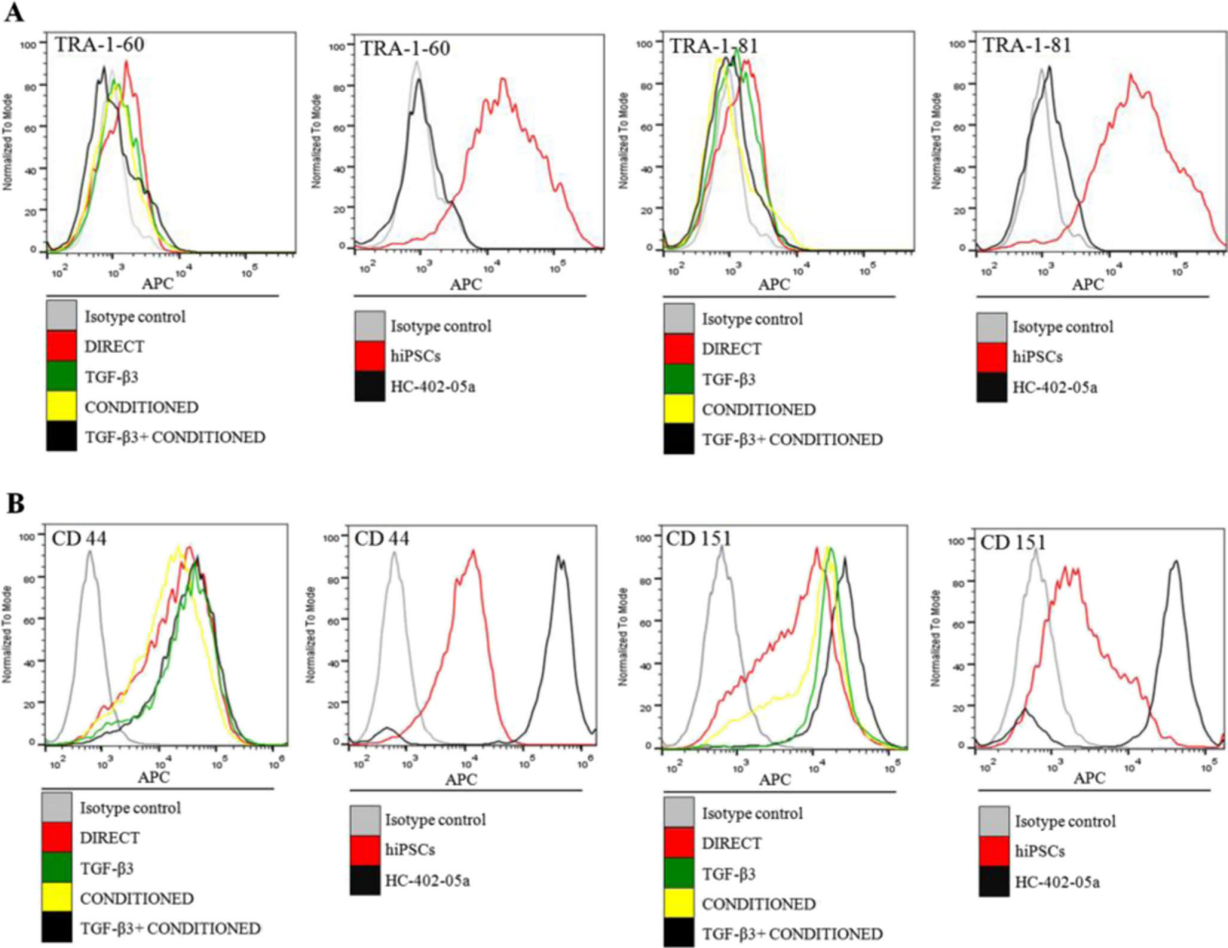
The lack of pluripotency markers TRA-1-60 and TRA-1-81 in the differentiated human-induced pluripotent stem cells (hiPSCs) was confirmed using flow cytometry (**a**). The chondrogenic markers CD44 and CD151 were observed in these cells

In all of the differentiated cells (i.e., from all four protocols), the pluripotency markers TRA-1-60 and TRA-1-81 were present but at a nearly undetectable levels. Only the positive control hiPSCs showed a high production of these proteins (Fig. 4a). The chondrocyte-like cells showed elevated levels of CD44 and CD151 proteins; however, the most visible shifts were present in the positive control (HC-402-05a) cell line (Fig. 4b).

The results obtained at the mRNA level are in compliance with results representing protein level. It is very difficult to unambiguously indicate the most efficient protocol of hiPSC chondrogenic differentiation. We have described four effective protocols of obtaining chondrocyte-like cells from hiPSCs because all obtained chondrocyte-like cells lost their pluripotent nature and first of all, reveal the features characteristic for mature chondrocytes. To selective the most appropriate and promising protocol for future clinical applications, there is a need to consider apart from gene and protein expression another factors such as the capacity of scale-up, culture and differentiation duration as well as overall costs. The most important issues are discussed in the next paragraph.

## Discussion

Current methods of differentiating hiPSCs into chondrocyte-like cells are inefficient and better methods are needed. In the present study, we evaluated and compared four different protocols used to differentiate hiPSCs into chondrocyte-like cells. The main aim was to evaluate and select the most effective and promising protocol(s) based on gene expression, protein analyses and time required for cell differentiation for obtaining chondrocyte-like cells from hiPSCs. We found that the two most efficient methods were the directed method (using defined GFs) and the medium conditioned with human cartilage chondrocytes. To our knowledge, neither of these protocols has been described previously yet both of these novel chondrogenic stimulators proved to be highly efficient. Below, we discuss our results in the context of the most recent data on chondrogenic differentiation of hiPSCs.

### Chondrogenic Differentiation in Vitro Using EBs in the Presence of TGF-β3 is the Most Common Method of Obtaining hiPSC-Derived Chondrocytes

Most established SC differentiation protocols include EB formation as a structure-containing mesoderm—the most important germ layer for induction of chondrogenesis—and further differentiation in the presence of TGF-β3 (the GF with the most chondrogenic potential). This approach appears to be the most effective and well-known [15]. However, an important drawback of this technique is that the use of EBs requires an additional step, thus increasing the time required for cell differentiation.

Villa-Diaz and co-workers [16] reported that hiPSCs cultured in xeno-free conditions can be efficiently differentiated into mesenchymal stem cells (MSCs) via EBs and subsequently into chondrogenic cells in a pellet culture in the presence of TGF-β3 (10 ng/ml). The high efficiency of this procedure was confirmed by the Safranin “O” staining and high expression of chondrogenic markers [16]. According to Lee and collaborators [17], hiPSCs can be efficiently differentiated into chondrocytes through EBs on fibronectin-coated plates for 14 days using a cocktail of defined GFs. However, note that this protocol requires a relatively large amount of GFs in high concentrations (up to 100 ng/ml), making this technique expensive and, therefore, diminishing its potential clinical application [17].

Li et al. [18] found that hiPSCs generated from human peripheral blood cells can form chondrocyte-like cells. In that study, the undifferentiated hiPSCs formed EBs, which were then transferred to gelatin-coated plates. After cellular confluence, cells were sorted by CD73 and CD105 double positivity and differentiated into chondrocytes in pellet culture in the presence of TGF-β1 (10 ng/ml) for 21 days [18]. MSC-like cells can also be obtained from hiPSCs by monolayer culture on type I collagen coatings that reflect the structure of physiological collagen, and by further differentiation in the chondrogenic medium in pellet culture [19]. Craft and co-workers [20] found that cells with features of streak and paraxial mesoderm obtained by hiPSC differentiation via EBs have a strong potential to create chondrocytes in high-density culture (micromass). In that study, chondrogenic differentiation was carried out in a serum-free chondrogenic medium in the presence of TGF-β3, forming stable cartilage tissue both in vitro and in vivo. Those authors also found that long-term supplementation with BMP-4 promotes the creation of undesirable hypertrophic chondrocytes capable of initiating endochondral ossification in vivo [20]. Ko et al. [21] compared the properties of chondrocytes obtained from hiPSCs with those generated from human bone marrow-derived MSCs (hBMMSCs). Both cell lines were differentiated via pellet culture in the presence of TGF-β3, but differentiation of the hiPSCs was preceded by EB formation. The hiPSC-derived chondrocytes presented both better glycosaminoglycan contents and better chondrogenic features. Furthermore, these cells had significantly reduced undesirable hypertrophic and osteogenic properties when compared to hBMMSC-derived chondrocytes [21].

Our results confirm previous reports [15–21] indicating that the use of EBs increases the efficiency of chondrogenic differentiation in hiPSCs. Importantly, we demonstrated that using a conditioned medium could replace (or enhance) the activity of exogenous TGF-β3. Indeed, hiPSCs differentiated via EBs in chondrogenic medium with TGF-β3 (TGF-β3 protocol), conditioned chondrogenic medium (COND protocol) or conditioned medium supplemented with TGF-β3 (COND + TGF-β3 protocol) (Fig. 1) all possess gene expression profiles (Fig. 2) and proteins (Figs. 3 and 4) that are characteristic of fully differentiated chondrocytes (e.g., type II collagen, COMP, CD44, SOX Trio).

### A Directed Method of hiPSC Chondrogenic Differentiation Constitutes a Novel, Promising Approach

Nejadnik and colleagues (2015) demonstrated that it is possible to generate chondrocyte-like cells from hiPSCs via in vitro differentiation without the need for EB formation. Firstly, they differentiated hiPSCs into MSC-like cells for 28 days and then differentiated these cells into chondrocytes in a high-density pellet culture in chondrogenic medium with TGF-β3 for another 21 days. Although the results obtained with this protocol were excellent, it is worth noting that it took 49 days to obtain the chondrocyte-like cells, thus making this time-consuming technique a mean candidate for routine differentiation [14].

Based on the previous mentioned work of Nejadnik et al. [14], our group has developed a novel differentiation protocol that does not require additional steps (e.g., EB formation) and only requires relatively small amounts of GFs. The findings reported in the present study suggest that this novel protocol could help to improve the process of obtaining chondrocyte-like cells. The cells obtained from this directed differentiation protocol presented chondrogenic features (Figs. 2, 3 and 4) that are comparable to cells differentiated through the well-known approach involving EB formation in the presence of TGF-β3. However, our protocol has the advantage of being more time- and cost-effective than currently available differentiation methods.

Regardless of the technique used to obtain chondrocytes from hiPSCs, the available evidence [22, 23] suggests that there may be a risk of tumorigenesis. Although implanted chondrocytes derived from hiPSCs can successfully regenerate cartilage tissue, they may also lead to tumor formation due to the development of immature teratomas from residual, undifferentiated hiPSCs; in addition, there is a risk of developing differentiated tumors, such as chondrosarcomas, originating from hiPSC-derived chondrocytes with genomic abnormalities [22, 23]. Chondrocytes derived from hiPSCs are therefore not without risk and more research into the genetic stability of these chondrocyte-like cells is needed.

## Conclusions

In this paper, we have described and compared four different protocols to obtain chondrocyte-like cells from hiPSCs. Our findings show that the addition of exogenous mesodermal and chondrogenic GFs as well as the use of a conditioned medium ensures that the chondrocyte-like cells present properties that are equivalent to, or even better than, those obtained from cultured human articular chondrocytes. Based on our data, it appears that the directed differentiation protocol presented in this study may offer an alternative to more expensive and slower in vitro hiPSC chondrogenic differentiation techniques. The benefit of direct differentiation is that it does not require additional steps (i.e., EB formation) and takes only 21 days, which is the fastest method reported to date for obtaining chondrocyte-like cells. Importantly, the use of conditioned medium may enhance the effect of exogenous TGF-β3 or even obviate the need for this GF, thus offering a novel and highly cost-effective method of hiPSC chondrogenic differentiation. Consequently, we believe the findings presented here may contribute to the development of more efficient methods of generating hiPSC-derived chondrocytes. Although we believe these findings represent an important breakthrough, more data are needed to better understand key aspects of the nature of chondrogenic hiPSC differentiation.

## Electronic supplementary material

Below is the link to the electronic supplementary material.

ESM 1 (DOCX 13 kb)

## References

[1] Robinson M, Chapani P, Styan T (2016). Functionalizing Ascl1 with novel intracellular protein delivery technology for promoting neuronal differentiation of human induced pluripotent stem cells. Stem Cell Reviews.

[2] Toh WS, Lee EH, Cao T (2011). Potential of human embryonic stem cells in cartilage tissue engineering and regenerative medicine. Stem Cell Reviews.

[3] Preitschopf A, Zwickl H, Li K (2012). Chondrogenic differentiation of amniotic fluid stem cells and their potential for regenerative therapy. Stem Cell Reviews.

[4] Fan Y, Wu J, Ashok P (2015). Production of human pluripotent stem cell therapeutics under defined xeno-free conditions: progress and challenges. Stem Cell Reviews.

[5] Guzzo RM, Scanlon V, Sanjay A (2014). Establishment of human cell type-specific iPS cells with enhanced chondrogenic potential. Stem Cell Reviews.

[6] Kulcenty K, Wróblewska J, Mazurek S (2015). Molecular mechanism of induced pluripotency. Contemporary Oncology (Pozn).

[7] Lee YK, Nq KM, Lai WH (2011). Calcium homeostasis in human induced pluripotent stem cell-derived cardiomyocytes. Stem Cell Reviews.

[8] Augustyniak E, Trzeciak T, Richter M (2015). The role of growth factors in stemcell-directed chondrogenesis: a real hope for damaged cartilage regeneration. International Orthopaedics.

[9] Lach, M., Trzeciak, T., Richter, M., et al. (2014). Directed differentiation of induced pluripotent stem cells into chondrogenic lineages for articular cartilage treatment. *Journal of Tissue Engineering,* 5:2041731414552701. 10.1177/2041731414552701 PMC4221915 25383175

[10] Barczak, W., Golusiński, P., Luczewski, L., et al. (2016). The importance of stem cell engineering in head and neck oncology. *Biotechnology Letters, 38*(10), 1665–1672. 10.1007/s10529-016-2163-7 PMC5010595 27341837

[11] Toh WS, Lee EH, Richards M, Cao T (2015). In vitro derivation of chondrogenic cells from human embryonic stem cells. Methods in Molecular Biology.

[12] Wróblewska, J. (2016) A new method to generate human induced pluripotent stem cells (iPS), and the role of the protein KAP1 in epigenetic regulation of self-renewal. Doctoral dissertation, http://www.wbc.poznan.pl/Content/373798/index.pdf.

[13] Suchorska WM, Lach MS, Richter M (2016). Bioimaging: an useful tool to monitor differentiation of human embryonic stem cells into chondrocytes. Annals of Biomedical Engineering.

[14] Nejadnik H, Diecke S, Lenkov OD (2015). Improved approach for chondrogenic differentiation of human induced pluripotent stem cells. Stem Cell Reviews.

[15] Yodmuang S, Marolt D, Marcos-Campos I (2015). Synergistic effect of hypoxia and morphogenetic factors on early chondrogenic commitment of human embryonic stem cell in embryoid body culture. Stem Cell Reviews.

[16] Villa-Diaz LG, Brown SE, Liu Y (2012). Derivation of mesenchymal stem cells from human induced pluripotent stem cells cultured on synthetic substrates. Stem Cells.

[17] Lee J, Taylor SE, Smeriglio P (2015). Early induction of a prechondrogenic population allows efficient generation of stable chondrocytes from human induced pluripotent stem cells. FASEB Journal.

[18] Li Y, Liu T, Van Halm-Lutterodt N (2016). Reprogramming of blood cells into induced pluripotent stem cells as a new cell source for cartilage repair. Stem Cell Research & Therapy.

[19] Liu Y, Goldberg AJ, Dennis JE (2012). One-step derivation of mesenchymal stem cell (MSC)-like cells from human pluripotent stem cells on a fibrillar collagen coating. PLoS One.

[20] Craft AM, Rockel JS, Nartiss Y (2015). Generation of articular chondrocytes from human pluripotent stem cells. Nature Biotechnology.

[21] Ko JY, Kim KI, Park S (2014). In vitro chondrogenesis and in vivo repair of osteochondral defect with human induced pluripotent stem cells. Biomaterials.

[22] Saito T, Yano F, Mori D (2015). Hyaline cartilage formation and tumorigenesis of implanted tissues derived from human induced pluripotent stem cells. Biomedical Research.

[23] Uto S, Nishizawa S, Takasawa Y (2013). Bone and cartilage repair by transplantation of induced pluripotent stem cells in murine joint defect model. Biomedical Research.

