# Supplementary material for: Comparison of Four Protocols to Generate Chondrocyte-Like Cells from Human Induced Pluripotent Stem Cells (hiPSCs)
Source: Stem Cell Rev. 2016 Dec 16;13(2):299–308. doi: 10.1007/s12015-016-9708-y (PMC5380716; doi:10.1007/s12015-016-9708-y)
Supplement: Supplementary file 1 — (DOCX 13 kb) [file 12015_2016_9708_MOESM1_ESM.docx]

| **Pluripotency genes** | **Primer** | **Sequence** | **Probe** |
| --- | --- | --- | --- |
| NANOG | Forward | atgcctcacacggagactgt | 31 |
|  | Reverse | aagtgggttgtttgcctttg |  |
| OCT4 | Forward | cttcggatttcgccttctc | 77 |
|  | Reverse | cttagccaggtccgaggat |  |
| SOX2 | Forward | tgcctctttaagactaggactgaga | 45 |
|  | Reverse | gccgccgatgattgttatta |  |
| E-CADHERIN | Forward | aagttttccaccaaagtcacg | 77 |
|  | Reverse | tgcttggattccagaaacg |  |
| **Chondrogenic genes** | **Primer** | **Sequence** | **Probe** |
| TYPE II COLLAGEN | Forward | ttctggagaccaaggtgctt | 18 |
|  | Reverse | ttccattagcaccatctttgc |  |
| SOX5 | Forward | ctccagcaacagatccaggt | 71 |
|  | Reverse | gggaggaggaatccttgc |  |
| SOX6 | Forward | gcttctggactcagcccttt | 67 |
|  | Reverse | ggagttgatggcatctttgc |  |
| SOX9 | Forward | ctcgccacactcctcctc | 77 |
|  | Reverse | cgcttcaggtcagccttg |  |
| NKX3.2 | Forward | gcacctggaggtgaaacc | 24 |
|  | Reverse | agctgccaggggacaagt |  |

Supplementary Table S1. RT- qPCR Primer sequence for pluripotency: NANOG, OCT4, SOX2, E-CADHERIN and chondrogenesis-associated: type II collagen, SOX5, SOX6, SOX9 genes. GAPDH (glyceraldehyde-3-phosphate dehydrogenase) was used as a house keeping gene.
